# Seventh time lucky—A case report of multiple radiofrequency ablations for right ventricular outflow tract tachycardia

**DOI:** 10.1002/ccr3.3394

**Published:** 2020-09-29

**Authors:** Gustav Mattsson, Peter Magnusson, Pekka Raatikainen

**Affiliations:** ^1^ Centre for Research and Development Uppsala University/Region Gävleborg Gävle Sweden; ^2^ Cardiology Research Unit Department of Medicine Karolinska Institutet Stockholm Sweden; ^3^ Department of Cardiology, Heart and Lung Center Helsinki University Hospital Helsinki Finland

**Keywords:** arrhythmia, cardiology, case report, electrophysiology, right ventricular outflow tract, ventricular tachycardia

## Abstract

Right ventricular outflow tachycardia initially refractory to radiofrequency ablation may be successfully treated after radiofrequency ablation at multiple sites. Repeated radiofrequency ablations as well as cooperation across borders with referral to an international center of excellence may be required in complicated cases

## Graphical abstract

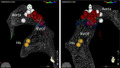

## 1 INTRODUCTION

Outflow tract ventricular tachycardia is the most common type of idiopathic ventricular tachycardia (VT) in those with structurally normal hearts. In approximately 80%‐90% of these patients, this originates from the right ventricular outflow tract (RVOT).^1^ In general, the prognosis of patients with RVOT VT is good.^2^ Treatment of RVOT premature ventricular complexes (PVCs) and VT includes antiarrhythmic medication and catheter ablation.^3^ Radiofrequency catheter ablation should be considered especially in those who have experienced syncope, very fast VT, or tachycardia‐induced cardiomyopathy or if symptoms remain despite medication.^2^ When idiopathic PVCs or VT originating from the RVOT can be mapped, catheter ablation is successful in 90%‐95% of patients with a recurrence risk of 5%.^3^

## 2 CASE REPORT

**Figure 1:**
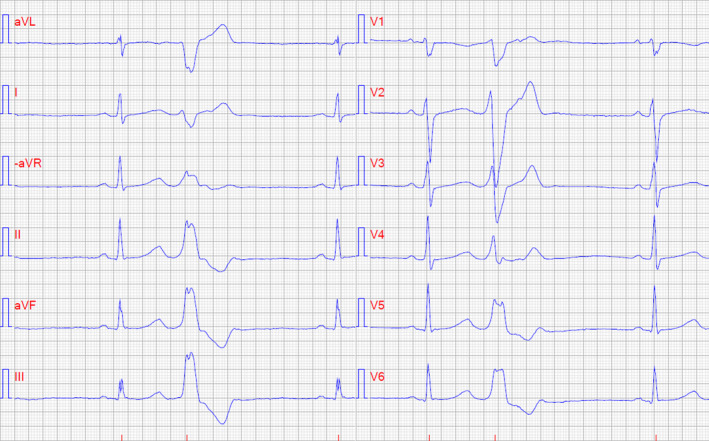
Twelve‐lead ECG with paper speed 50 mm/s showing sinus rhythm with premature ventricular complexes in bigeminy. Premature ventricular complexes show inferior axis and left bundle branch morphology indicative of an origin in the basal part of the right ventricle

A 15‐year‐old girl was referred to a pediatrician due to repeated episodes of palpitations, dizziness, and presyncope (Table 1). Seven months prior, she experienced chest discomfort and palpitations followed by syncope while walking. She had a history of post‐traumatic stress syndrome but was free from pharmacological therapy. Family history with regard to cardiac disease was unremarkable. Twelve‐lead ECG was normal except for frequent monomorphic PVCs (Figure 1). Echocardiography ruled out structural heart disease. A 24‐hour ambulatory ECG showed sinus rhythm with more than 20,000 monomorphic PVCs with left bundle branch block morphology in V_1_‐V_2_ and electrical inferior frontal axis between 90° and 120°. During a bicycle exercise testing, the number of PVCs decreased when heart rate elevated. A Holter recording four months later revealed 86 episodes of monomorphic nonsustained VT (NSVT) with the same morphology as the PVCs in the initial Holter recording were detected. There were no late potentials in a signal‐averaged ECG. The patient had no further syncope but continued to experience palpitations and metoprolol 100 mg once daily was started to alleviate symptoms. After this, she was lost to follow‐up for four years due to social factors.

**Table 1:** Timeline

Time	Events
2003 (September)	First unexplained syncope
2008 (April)	Electrophysiology study
2011 (September)	Electrophysiology study
2012 (June)	Attempted ablation
2012 (June)	Attempted ablation
2012 (October)	Electrical storm
2012 (October)	Attempted ablation
2012 (November)	Electrophysiology study, Tampere
2012 (November)	Successful ablation, Tampere

She was mainly asymptomatic during these years but at an age of 18 she experienced a single episode of palpitations during pregnancy. Her heart rate was irregular but no ECG was taken. A cesarean section was performed without complications. Two years later during her second pregnancy, she was referred to the cardiology clinic due to frequent palpitations. During a 24‐hour ambulatory ECG monitoring, the longest NSVT was 12 beats at a rate of 200 beats per minute. After childbirth, she was assessed at a tertiary center with cardiac magnetic resonance that was normal except for an area that was 2 mm in diameter in the right ventricle with increased adipose tissue. This was deemed not diagnostic for arrhythmogenic right ventricular cardiomyopathy, and no arrhythmias were inducible in an electrophysiological study (EPS).

**Figure 2:**
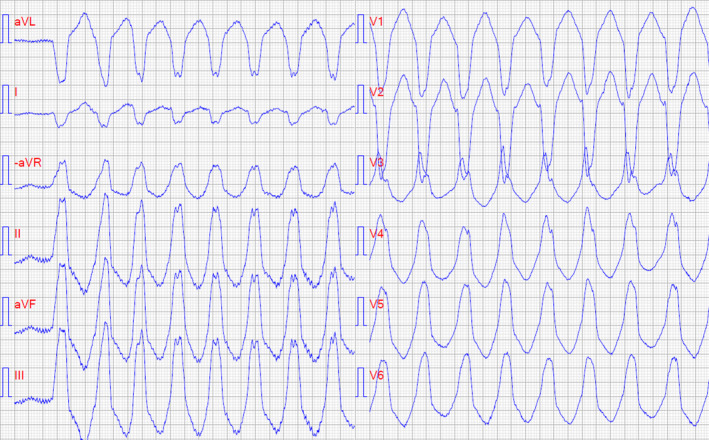
Twelve‐lead ECG with paper speed 50 mm/s showing monomorphic ventricular tachycardia (200 beats per minute)

Palpitations continued despite beta‐blocker therapy, and three years later, the patient received an insertable cardiac monitor. A few months later, she had recurrent syncope and several episodes of rapid NSVT up to 33 beats were revealed. EPS was repeated and a short run of VT arising from the RVOT was induced. This was not reproducible and ablation could not be performed. Instead, flecainide 100 mg twice daily and sertraline 50 mg once daily was initiated due to palpitations and anxiety, respectively. Arrhythmia symptoms were ameliorated by flecainide, but the medication had to be stopped due to visual adverse effects. Thereafter, the patient was referred to another tertiary center for a third EPS and ablation. However, 5 days later recurring NSVT returned. Intravenous amiodarone decreased the frequency and duration of the VTs, but the patients remained highly symptomatic, and she was transported with airborne ambulance back to the tertiary center for reablation. Despite several ablations in the RVOT, the arrhythmia did not stop and antiarrhythmic medication was reinitiated. Four months later, she presented to the emergency department with electrical storm due to repeated episodes of sustained monomorphic VT (Figure 2), which terminated following intravenous amiodarone.

**Figure 3:**
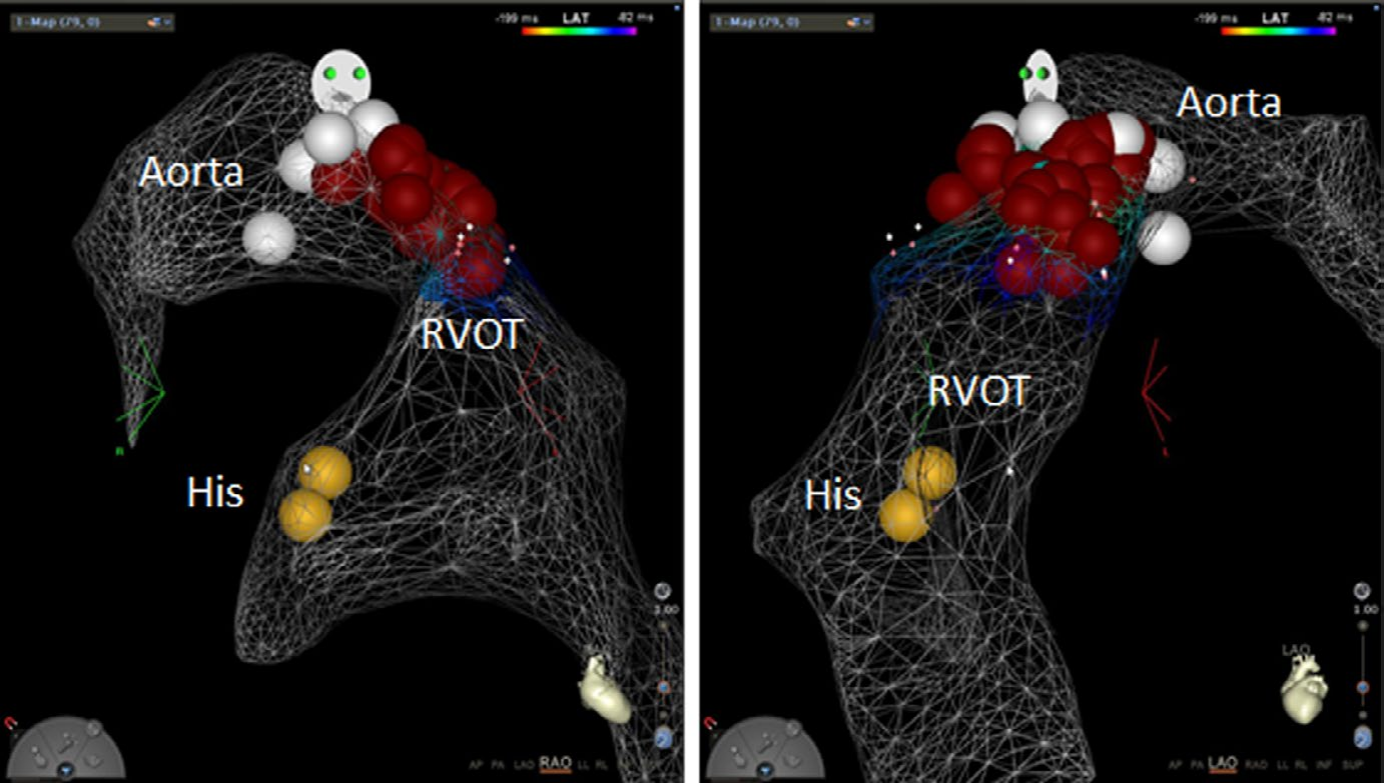
Electroanatomic map (Carto™) of the right ventricular outflow tract and aortic root in the right anterior oblique (RAO) and left anterior oblique (LAO) view. The His bundle position is marked with orange dots. Catheter ablation was performed first in the aortic root at the junction of the right and noncoronary cusp (white dots) using remote magnetic navigation (Stereotaxis™). The ventricular tachycardia stopped and the number of the premature ventricular complexes (PVCs) was reduced, but they were not eliminated completely despite several ablations in the aortic root. Additional ablations (red dots) in the right ventricular outflow tract (RVOT) in the same area eliminated PVCs and since the ablation she has had no ventricular tachycardia

After washout of antiarrhythmic medications, a fifth EPS was performed with ablation in the RVOT but arrhythmia remained after the procedure. It was decided to refer the patient to a tertiary arrhythmia center in Finland. In the initial EPS in Tampere University Hospital, neither PVCs nor any VT was detected and no ablation was performed. However, during the following night the patients had frequent PVCs and multiple VTs. She was taken back for EPS in the next morning. Electroanatomical mapping using remote magnetic navigation revealed slightly earlier activation in the aortic root between the right and noncoronary cusp than in the RVOT area. Pace map in this site was excellent. Following ablation in this site, VT stopped but the PVCs did not disappear despite multiple ablations. Mapping of the distal coronary sinus and left ventricular outflow tract was not successful, whereas remapping of the RVOT revealed early activation and good pace map. Upon ablation in the RVOT (Figure 3), PVCs disappeared and no VT was inducible at programmed ventricular stimulation. The patient was discharged two days later with no symptoms. During the seven‐year follow‐up period after the last ablation, she has had sporadic PVCs from another focus but no sustained or nonsustained VT has been detected by the insertable cardiac monitor or by ECG.

## 3 DISCUSSION

Evaluation of patients with frequent PVCs or VT originating from the right ventricle should include prompt evaluation regarding arrhythmogenic right ventricular cardiomyopathy. In the current case, 12‐lead ECG, signal‐averaged ECG, repeated echocardiography and cardiac magnetic resonance imaging provided no evidence structural heart disease.^4^ Hence, she was diagnosed with idiopathic RVOT tachycardia. RVOT tachycardia is usually benign, but like in this case, it can occasionally lead to intolerable symptoms and frequent VT episodes.

Due to the close proximity of the aortic sinus cusps and the RVOT, it is hard to differentiate ventricular arrhythmias arising from these structures as both of them can present with inferior axis and left bundle branch block morphology.^5^ Guidelines recommend that for ventricular arrhythmia with left bundle branch block morphology and inferior axis, mapping should start with the RVOT. If ablation in the RVOT fails, it is recommended to map also the left ventricular outflow tract and sinus of Valsalva as well as the distal coronary sinus, and in some cases also epicardial mapping may be needed.^6^ In this case, initial ablation in the RVOT seemed to be successful but the arrhythmia reoccurred soon after the procedure and multiple reablation attempts failed. Hence, it is likely that the arrhythmia substrate was complex and catheter ablation both in the aortic root and in the corresponding area in the RVOT was required to eliminate the PVCs and VT.

In the last ablation, remote magnetic navigation (Stereotaxis™) was used for mapping and ablation. In our experience, mapping of the outflow tract area with remote magnetic navigation is feasible and catheter‐induced PVCs are less common than during manual catheter manipulation. A retrospective study has shown similar acute and chronic success rates between remote magnetic navigation and manually controlled catheter ablation for RVOT VT.^7^ Finally, our case indicates that close collaboration between centers across borders is invaluable for the management of complex arrhythmias.

In complex cases of RVOT and other arrhythmias, repeated ablation procedures as well as cooperation across borders may be required for a successful outcome. It is possible that in cases like this with failed manual catheter ablations, an alternative approach using remote magnetic navigation may be effective.

## CONFLICT OF INTEREST

GM has received speaker's fee from Alnylam, MSD, and Internetmedicin. Outside this project: PM has received speaker's fees or grants from Abbott, Alnylam, AstraZeneca, Bayer, Boehringer‐Ingelheim, Internetmedicin, Lilly, MSD, Novo Nordisk, Octopus Medical, Orion Pharma, Pfizer, Vifor Pharma, and Zoll. PR has acted as consultant for Biosense Webster and Stereotaxis in management of complex atrial and ventricular arrhythmias. PR has received speaker´s fees from Abbott, Bayer, Biosense Webster, Biotronik, BMS, Pfizer, and Stereotaxis.

## AUTHOR CONTRIBUTIONS

All authors were involved in compilation of data and manuscript preparation. G.M: wrote the first draft of the manuscript. PM and P.R: were the senior consultants involved in the overall management of the patient and catheter ablation procedure, respectively. Approval of the authors: All authors approved the final version of the case report for submission to the Clinical Case Reports.

## ETHICAL APPROVAL

Not applicable. The patient gave written informed consent to the publication of this case report.
